# From Coffee Coproduct to Functional Ingredient: Coffee Silverskin Flour as a Sustainable Fat Replacer in Ice Cream and Its Impact on Nutritional, Physicochemical, and Sensory Acceptance

**DOI:** 10.3390/foods15132355

**Published:** 2026-07-02

**Authors:** Laura Candela-Salvador, Juana Fernández-López, Luis Noguera-Artiaga, José Ángel Pérez-Álvarez, Raquel Lucas-González, Manuel Viuda-Martos

**Affiliations:** 1 Innovations in Food Products (IPOA) Research Group, Institute for Agri-Food and Agri-Environmental Research and Innovation, Miguel Hernández University (CIAGRO-UMH), 03312 Orihuela, Alicante, Spain; laura.candela03@goumh.umh.es (L.C.-S.); j.fernandez@umh.es (J.F.-L.); ja.perez@umh.es (J.Á.P.-Á.); raquel.lucasg@umh.es (R.L.-G.); 2 Food Quality and Safety (CSA) Research Group, Institute for Agri-Food and Agri-Environmental Research and Innovation, Miguel Hernández University (CIAGRO-UMH), 03312 Orihuela, Alicante, Spain; lnoguera@umh.es

**Keywords:** coffee, waste valorization, coproducts, ice cream, nutritional enrichment, functional foods

## Abstract

This study evaluates the feasibility of using coffee silverskin flour (CSF) as a milk cream replacer in full-fat ice cream to enhance its nutritional and functional profile. Two formulations were developed by replacing milk cream with CSF at levels of 15.78% (SSIC25) and 31.56% (SSIC50). Results showed a significant reduction (*p* < 0.05) in fat content, decreasing from 10.14 g/100 g in the control sample to 5.91 g/100 g in SSIC50. Concurrently, total dietary fiber increased significantly from 3.22 to 7.34 g/100 g. The incorporation of CSF also enriched the mineral profile, with calcium and potassium levels increasing by 27.77% and 38.56% in SSIC50, respectively. Bioactive compounds were notably enhanced; caffeine content reached 335.25 mg/100 g, and caffeic acid derivatives reached 69.51 mg/100 g in the highest substitution level. Physicochemical analysis revealed that overrun increased significantly from 29.2% to 52.17%, while the melting rate remained unaffected. Sensory evaluation indicated that although CSF increased bitterness and grittiness, the SSIC25 formulation maintained favorable consumer acceptance scores (>6.0). In conclusion, coffee silverskin flour serves as a sustainable, health-promoting ingredient for frozen desserts, with a 15.78% replacement level identified as the optimal threshold for balancing nutritional improvement and sensory acceptance.

## 1. Introduction

Ice cream is one of the most widely consumed frozen dairy products worldwide, appreciated for its sensory attributes, including creamy texture, sweetness, and flavor complexity [1]. Its formulation typically includes several ingredients such as lipids, proteins, hydrocolloids, carbohydrates, stabilizers, and emulsifiers dispersed in the formulation matrix. Further processing involves homogenization, aging, and freezing processes that determine its final microstructure and quality attributes [2]. Despite its popularity, conventional ice cream has a high fat content, which represents a significant nutritional limitation. Ice cream consumption increases the intake of saturated fatty acids, which has been associated with chronic diseases such as cardiovascular disorders and obesity. Accordingly, the global demand for reduced-fat dairy products has experienced sustained annual growth of more than 4% and is projected to continue expanding at a compound annual growth rate close to 5% over the coming decade (2025–2035) [3]. This growing consumer preference for healthier dairy foods has stimulated the development of innovative formulations incorporating functional ingredients capable of improving nutritional quality while maintaining desirable physicochemical and sensory properties. However, fat fraction plays a key technological role by enhancing mouthfeel, creating a stable fat network that traps air bubbles and contributes to a smooth texture [4], and contributing to melting resistance. Its reduction often leads to defects such as increased ice crystal size, poor melting behavior, weak structure, and diminished creaminess [5].

It should be borne in mind that reducing the fat content in ice cream is not an easy task. In recent years, increasing attention has been directed toward the use of plant-based ingredients, particularly powder and flours obtained from fruits, vegetables, and their coproducts which have a high content of dietary fibers and bioactive compounds, as fat replacers in ice creams [6,7,8]. These ingredients, including peels, seeds, pomace, and pulp residues, may replicate some of the physicochemical roles of fat while improving the nutritional profile of the final product [9]. The mechanism by which flours or powders obtained from fruit coproducts act as fat replacers in ice creams is mainly related to their high fiber content and associated water-binding capacity [10]. Insoluble fibers contribute to the formation of a particulate network, while soluble fibers increase viscosity and stabilize the continuous phase [11]. This double techno-functionality could help to simulate the mouthfeel and structuring effects of fat. Additionally, the presence of phenolic compounds may interact with proteins and polysaccharides, further influencing the rheological properties of the ice cream mix [12]. On the other hand, the addition of coproducts into food formulations aligns with the principles of a circular economy and sustainability, transforming waste streams into value-added ingredients [13].

Coffee silverskin, a coproduct generated during coffee roasting, is generated in large volumes and, due to its composition rich in bioactive compounds (with polyphenols such catechins, methylxanthines such as caffeine, and dietary fiber [14]), could be used as a fat substitute in ice cream production. In this sense, the use of coffee silverskin in the formulation of several food products has been widely studied, particularly in bakery [15,16], cereal-based [17,18], and meat [19,20] products. Its incorporation has been associated with increased fiber content and enhanced antioxidant capacity, although it may also influence color and flavor due to its characteristic roasting process.

In this context, the present study aimed to evaluate the feasibility of using coffee silverskin as a fat replacer in full-cream ice cream formulations, with the objective of developing a nutritionally enriched dairy product with potential health benefits. Although coffee silverskin has been extensively investigated as a sustainable food ingredient, to the best of our knowledge, its potential as a fat replacer in ice cream formulations to reduce fat content has not yet been explored. Therefore, sensory and physicochemical analyses were performed to comprehensively characterize the resulting ice creams.

## 2. Materials and Methods

### 2.1. Ice Cream Ingredients

Whole milk (fat 3.6 g/100 mL; proteins 3.1 g/100 mL; carbohydrates: 4.6 g/100 mL), milk cream (fat 38 g/100 mL; proteins 1.8 g/100 mL; carbohydrates: 2.7 g/100 mL), skim milk powder (fat 1 g/100 g; proteins 36.3 g/100 g; carbohydrates: 54 g/100 g), and sugar were obtained from a local market in Orihuela, Spain. Inulin Orafti^®^HP was supplied by Beneo (BENEO GmbH; Mannheim; Germany), while the soy lecithin was provided by El Granero (El Granero S.L.; Madrid, Spain). The silverskin samples were obtained from coffee (*Coffea canephora*) beans cultivated in India and supplied by the CoffeeShop company (Elche, Spain). These coffee beans were subjected to a light roasting process at 195 °C for 12 min. The silverskin samples were ground in a Titanmill 300 coffee grinder (Cecotec, Valencia, Spain) to obtain the flour, with a particle diameter lower than 210 µm. Afterward, the flour was stored in dark vacuum bags until use. The flours obtained had a total dietary fiber of 74.30 g/100 g, total free and bound polyphenolic compounds of 19.71 and 219.39 µg/g, respectively, while the caffeine content was 4.37 mg/g [14]. In reference to techno-functional properties, the CSF showed a water-holding capacity, oil-holding capacity, and swelling capacity of 4.20 g water/g flour, 2.69 g oil/g flour, and 4.53 mL/g flour [14].

### 2.2. Ice Cream Elaboration

The control full-fat ice cream, as well as the ice cream samples with the addition of silverskin flour as a fat replacer, were elaborated following the recommendations of Balivo et al. [21], with some modifications. For that, all ingredients were weighted individually according to the formulations shown in Table 1.

**Figure 1:**
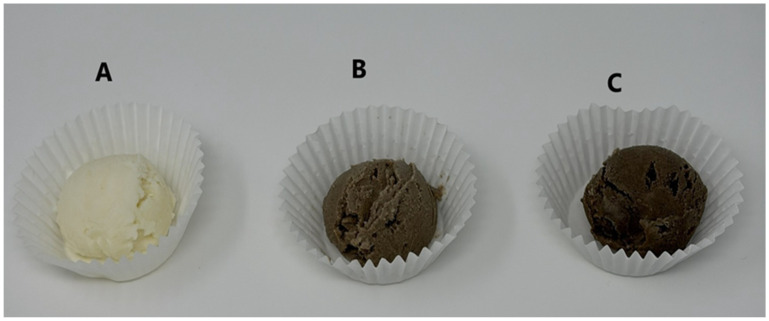
Control ice cream and reformulated ice creams added with coffee silverskin flour as fat replacers. (**A**) Control sample (CS); (**B**) ice cream with 15.78% cream replaced by coffee silverskin flour (SSIC25); (**C**) ice cream with 31.56% cream replaced by coffee silverskin flour (SSIC50).

The control formulation was elaborated without the substitution of milk cream for coffee silverskin flour. In contrast, formulations designated as SSIC25 and SSIC50 incorporated coffee silverskin flour as a partial fat replacer at substitution levels of 15.78% and 31.56%, respectively. For the preparation of the reformulated samples, milk and coffee silverskin flour were first homogenized using an IKA T25 UltraTurrax homogenizer (IKA^®^-Werke GmbH & Co., Staufen, Germany) for 5 min at 14,000 rpm. Then, the milk cream and the previously mixed dry ingredients were added to the mixture, which was mixed using an homogenizer IKA T25 for 5 min at 14,500 rpm and pasteurized at 72 °C for 25 min. The ice cream mixes were maintained at 4 °C for 24 h. After this, the samples were aerated using the IKA T25 UltraTurrax homogenizer for 1 min at 400 rpm, and the ice cream was made using a Caso ice cream machine (Caso Design, Arnsberg, Germany) at −18 °C ± 1 °C for 28 min ± 3 min. The control and reformulated ice creams (Figure 1) were placed in plastic trays and hardened at −20 °C for 18 h prior to analysis.

### 2.3. Chemical Composition

The assessment of the chemical composition of the ice creams (moisture, protein, fat, ash, and total dietary fiber content) was carried out following the recommendations of the Association of Official Analytical Chemistry [22]. The results were expressed as g/100 g of ice cream. The mineral profile of the control and reformulated ice creams was analyzed by means of Inductively Coupled Plasma Mass Spectrometry (ICP-MS) following the specifications of Candela-Salvador et al. [14]. The results were expressed as mg/100 g of ice cream.

### 2.4. Physicochemical Properties

The ice cream water activity, pH, and color were determined based on the following procedures. The ice cream pH value was measured using a pH meter Sension + pH31 (HACH, Derio, Spain) combination electrode. The water activity (aw) was determined using a hygrometer LabSwift-aw (Novasina AG, Lachen, Switzerland) at 25 °C.

The color was determined using the CIEL*a*b* color space with a CM-700 spectrophotometer (Minolta Camera Co., Osaka, Japan), with D_65_ as the illuminant and 10° as the observed standard. The following color coordinates were determined: lightness (L*), redness (a*), and yellowness (b*). From these coordinates, the psychophysics parameters of hue (h) and chroma (C*) were determined using Equations (1) and (2). The total color difference (ΔE) of each reformulated ice cream (I), with reference to the control ice cream (C), as well as the browning index were assessed by means of Equations (3) and (4), respectively.

(1) $$C^{*}=\sqrt[{2}]{{{{{(a}^{*})}^{2}+(b}^{*})}^{2}}.$$

(2) $$h=arctg\frac{b^{*}}{a^{*}}.$$

(3) $${\Delta E}^{*}=\sqrt[{2}]{{\left(L_{I}^{*}-L_{C}^{*}\right)}^{2}+{\left(a_{I}^{*}-a_{C}^{*}\right)}^{2}+{\left(b_{I}^{*}-b_{C}^{*}\right)}^{2}}.$$

(4) $$Browning\;index=\frac{\left(\frac{\left(a^{*}+1.75L^{*}\right)xa^{*}}{5.645L^{*}+a^{*}-3.012b^{*}}\right)-0.31}{0.17}.$$

The overrun value of the control ice cream and reformulated ice creams added with coffee silverskin flour as fat replacers were measured using a 150 mL cup. Ice cream mixes (150 mL) and ice cream samples (150 mL) were weighed, and the overrun (OR) was calculated using Equation (5), as suggested by Muse and Hartel [23].

(5) $$\%Overrun=\frac{Weight\;of\;ice\;cream\;mix-Weight\;of\;ice\;cream}{Weight\;of\;ice\;cream}.$$

The melting rate of the control ice cream and reformulated ice creams added with coffee silverskin flour as fat replacers was assessed using an ice cream sample (200 mL) which was stored at −21 °C for 4 days [24]. Ice cream samples were transferred onto a stainless-steel mesh screen (1 mm aperture) and maintained at room temperature (22 °C) until complete melting occurred. The mass of the melted fraction was determined at 10 min intervals. Melting behavior was evaluated by plotting the percentage of melted ice cream against time, and the melting rate (g/min) was calculated from the slope of the linear region of the curve.

### 2.5. Aroma Profile

#### 2.5.1. HS-SPME Preparation for Volatile Compound Extraction

Volatile organic compounds were extracted using HS-SPME following the recommendations of Welty et al. [25], with some modifications. For that, 5 g of control or reformulated ice cream was weighed into a 20 mL vial. Afterward, 3 mL of 30% (*w*/*v*) sodium chloride solution was incorporated to promote the transfer of volatile constituents into the headspace, followed by vortex mixing for 3 min. Volatile compounds were extracted using a 1 cm solid-phase microextraction (SPME) fiber coated with divinylbenzene/carboxen/polydimethylsiloxane (DVB/CAR/PDMS, 50/30 µm film thickness). Headspace-SPME extraction was conducted at 50 °C for 50 min while maintaining constant agitation at 450 rpm. All extraction procedures were carried out with a Shimadzu AOC-6000 Plus autosampler (Shimadzu Corporation, Kyoto, Japan). Immediately after extraction, the fiber was transferred to the gas chromatograph injection port for thermal desorption of the adsorbed analytes.

#### 2.5.2. HS-SPME Preparation for Volatile Compound Extraction

Gas chromatographic separation was conducted in a GC/MS Shimadzu model TQ8040NX (Shimadzu Corporation, Kyoto, Japan) by means of a SAPIENS X5MS capillary column (30 m × 0.25 mm i.d. × 0.25 µm film thickness; Teknokroma, Barcelona, Spain). The oven temperature program was as follows: initial temperature 40 °C, increased to 130 °C at 2 °C/min, then ramped to 180 °C at 10 °C/min, and held for 5 min. Finally, the temperature increased to 280 °C at 20 °C/min. The carrier gas used was helium at 60 mL/h. Injection was made in split-less mode at 220 °C. Mass spectrometric analysis was performed with a Shimadzu Corporation Shimadzu TQ8040 NX operating under electron ionization conditions at 70 eV. The ion source and quadrupole temperatures were maintained at 230 °C and 280 °C, respectively. Data acquisition was conducted over an m/z interval of 35–500 with a scan speed of 0.1 scans/s.

Volatile compound identification was achieved with two procedures: (i) calculation of retention indices based on analysis of a C8–C24 n-alkane standard mixture (Sigma-Aldrich, Steinheim, Germany), and (ii) matching of experimental mass spectra with reference spectral databases (>90%) [26].

### 2.6. Polyphenolic Profile Analysis

#### 2.6.1. Polyphenolic Compound Extraction

Polyphenolic compound extraction from reformulated ice creams added with coffee silverskin as a fat replacer was conducted following the methodology reported by Lucas-González et al. [27], with slight modifications. For that, the control and the reformulated ice creams (5 g) were subjected to two extraction steps in sequence; first with 50 mL methanol-water (8:2 *v*/*v*) and then with 50 mL acetone-water (7:3 *v*/*v*). In each extraction procedure, samples were sonicated (15 min in ultrasonic bath, without control temperature) and centrifugated (3000× *g*, 10 min and 4 °C). Both collected supernatants were combined and then evaporated to dryness in a rotary evaporator BUCHI-300 (BUCHI Ibérica S.L.U., Barcelona, Spain). The extract was resuspended in water and then passed through a C-18 CHROMAFIX^®^ Sep-Pak cartridge (Macherey-Nagel, Düren, Germany). The polyphenolic compounds were collected in methanol-formic acid (99:1 *v:v*). The extracts obtained were maintained at −21 °C until high performance liquid chromatography (HPLC) analysis.

#### 2.6.2. High Performance Liquid Chromatography (HPLC) Analysis

The polyphenolic profile of reformulated ice creams added with coffee silverskin as a fat replacer was carried out with a Hewlett-Packard HPLC series 1200 instrument (Agilent, Waldbronn, Germany) equipped with a UV-DAD detector and a Mediterranea Sea-18 column (Teknokroma, Barcelona, Spain), following the method proposed by Genskowsky et al. [28]. Quantification was performed using calibration curves of authentic standards. For compounds where a specific standard was not available, semi-quantification was conducted using a representative compound from the same chemical subfamily. The values were expressed as mg/100 g of ice cream.

### 2.7. Caffeine Content

The caffeine concentration of the control and reformulated ice creams added with coffee silverskin was determined using the methodology described by Grillo et al. [29]. The extracts obtained in Section 2.6.1. were analyzed by HPLC using the methodology reported by Candela-Salvador et al. [14]. The results were expressed as mg of caffeine/100 g of ice cream.

### 2.8. Sensory Analysis

A hedonic sensory evaluation was conducted with 95 consumers (33% male, 67% female), primarily consisting of students and faculty members. The assessment took place in a single session at the Sensory Analysis Laboratory of CIAGRO-UMH (Orihuela, Spain). Before starting the study, all participants were informed about the specific characteristics of the product to be evaluated as well as the purpose and procedures of the analysis. Written informed consent was obtained from each participant. The study protocol was approved by the Responsible Research Office of Miguel Hernández University (OIR-Reg. 240504113706, Ref. ADH.RTA.MVM.LCS.24, UMH, Elche, Alicante, Spain). The participants were asked to score the sensory attributes, including overall acceptability, aroma, color, taste, sweetness, bitterness, and grittiness, which were evaluated using a nine-point hedonic scale (1 = dislike extremely, 5 = neither like nor dislike, and 9 = like extremely). Prior to evaluation, the ice cream samples (4 days after storage at −18 °C) were taken from a freezer and allowed to temper at room temperature for 8 to 10 min to achieve a temperature of −10 °C. The different ice cream samples were served in clear 30 mL plastic cups. To help cleanse the palate between samples, participants were provided with crackers and water.

### 2.9. Statistical Analysis

The ice creams were elaborated in three independent batches (replicates) on separate days. The results obtained were presented as mean ± standard deviation. The data were evaluated by one-way analysis of variance (ANOVA), and if statistically significant differences were found, a Tukey post hoc test was performed at a 95% significance level (*p* < 0.05). Data were analyzed using the statistical package SPSS Statistics v.26 (IBM Corp., Armonk, NY, USA).

## 3. Results and Discussion

### 3.1. Chemical Composition

Table 2 shows the chemical composition of the control ice cream and reformulated ice creams added with coffee silverskin flour. The fat replacement in ice cream with coffee silverskin flour led to statistically significant changes (*p* < 0.05) in the total content of the main components of ice cream. In this sense, the moisture and fat content decreased progressively as the level of substitution with coffee silverskin flour increased. Specifically, moisture content was reduced from 61.89 g/100 g in the control ice cream to 58.15 g/100 g in SSIC50. These findings are consistent with those reported by Balivo et al. [21], who observed a decrease in moisture content in low-fat ice creams formulated with whipped chickpea aquafaba as a fat replacer as the substitution level increased. The observed reduction in moisture content can be attributed to the compositional and structural changes induced by fat replacement. The incorporation of coffee silverskin flour increases the total solid content of the formulation, particularly due to its high fiber content [14], thereby reducing the relative proportion of water. Additionally, the reduction in water content can be partially explained by the lower amount of cream incorporated into the formulation, as cream contains 57.4 g of water per 100 g of product. Consequently, replacing cream results in a lower water input into the final ice cream formulation. Regarding fat content (Table 2), replacement with coffee silverskin flour resulted, as expected, in a reduction in this parameter, reaching decreases of 21.10% and 41.71% for SSIC25 and SSIC50, respectively (*p* < 0.05). These results agree with those reported by Moolwong et al. [30], who described reductions in fat content ranging from 12% to 28% in ice creams where part of the cream (10% and 30%) was replaced with avocado pulp. Similarly, Zuniga Moreno et al. [31] reported that the use of sugarcane bagasse as a partial fat replacer at levels of 15%, 20%, and 25% led to reductions in fat content ranging from 53.40% to 81.99%.

In contrast, protein and ash contents showed the opposite trend, increasing as fat was replaced by coffee silverskin flour. In this sense, protein content increased by 17.74% and 32.95% for SSIC25 and SSIC50, respectively (*p* < 0.05), with respect to the control ice cream, while ash content (Table 2) varied from 0.87 g/100 g in the control sample to 1.36 g/100 g in SSIC50, with statistically significant differences observed among the samples (*p* < 0.05). This fact might be attributed to the protein and mineral content of the added silverskin flour used to replace fat in the ice cream. Similar trends have been reported in reformulated ice creams enriched with plant-derived ingredients, where the incorporation of solid-rich matrices leads to higher protein levels and mineral content due to the redistribution of total solids within the system. Khalil et al. [32] showed that low-fat ice cream added with sweet lupine flour as a fat replacer increased the protein and ash content compared with full-fat ice cream, whereas Elkot et al. [33] reports similar results in low-fat ice creams elaborated using camel milk and added with different amounts of heart of date palm (4%, 8%, and 12%) as fat replacers.

With reference to the fiber content (Table 2), the addition of coffee silverskin flour increased the dietary fiber content (*p* < 0.05) in a concentration-dependent manner. Thus, the total dietary fiber content increased by 60.86% and 127.95% for SSIC25 and SSIC50, respectively (*p* < 0.05), with respect to the control ice cream. This increase may be due to the fiber content present in the coffee silverskin flour, which was 74.44 g/100 g [14]. Beyond its fundamental role in enhancing the techno-functional properties and stabilizing the air–cell matrix, such levels are critical for elevating the nutritional profile of dairy-based matrices [34]. The incorporation of flours with a high content of dietary fiber, such as at these concentrations, effectively modulates the glycemic response, mitigating the rapid postprandial glucose spikes typically associated with desserts with a high sugar content such as ice creams [35]. Additionally, these formulations have a high dietary fiber content that may enable them to act as effective prebiotic matrices by improving probiotic viability. They may also modulate metabolites associated with gut microbiome balance and intestinal homeostasis [36,37].

The mineral profiles of the control and reformulated ice creams added with coffee silverskin flour are shown in Table 3. The addition of coffee silverskin flour as a fat replacer caused significant changes (*p* < 0.05) in the mineral profile compared with the control sample. Thus, the calcium, copper, iron, potassium, magnesium, and zinc content increased significantly (*p* < 0.05) in the reformulated samples compared to the control ice cream, while the concentration of sodium and phosphorus decreased (*p* < 0.05) with the use of coffee silverskin flour as a fat replacer compared with the control ice cream.

The results agree with the mineral content present in the coffee silverskin flour, which showed high levels of potassium, calcium, and magnesium [14]. It is important to highlight that the calcium and potassium contents of the reformulated ice creams increased by 23.85% and 33.66%, respectively, in SSIC25 in comparison with the control sample, and by 27.77% and 38.56%, respectively, in SSIC50 compared with the control sample. These increases in calcium and potassium are nutritionally relevant, as they contribute to improving the dietary intake of essential minerals commonly under-consumed in Western populations [38]. Thus, 100 g of SSIC25 ice cream provides, for adults, around 20.5% of the recommended daily intake (RDI) for calcium, and 12.9, 7.8, and 8.9% of the RDI for iron, potassium, and magnesium, respectively. In the case of SSIC50, 100 g of ice cream provides, approximately, 21% of the RDI of calcium and 23%, 8%, and 10% of the RDI for iron, potassium and magnesium.

From a functional perspective, the results confirmed that the incorporation of coffee silverskin flours not only enhances the nutritional profile of ice cream but also maintains its suitability as a dairy-based delivery matrix for bioavailable minerals, supporting its potential as a functional food product. These results agree with those reported by Yangilar [39], who reported that the addition of peach peel and pulp, at 1 and 2%, in ice creams increased the rates of calcium, potassium, and magnesium. Similarly, Nasr [40] reported that the fortification of ice cream with 5, 10, 15% of sapota fruit pulp and sucrose significantly increased the concentration of calcium and potassium.

### 3.2. Physicochemical Properties

The physicochemical properties of the control ice cream and formulations in which fat was partially replaced by coffee silverskin flour are presented in Table 4. The water activity decreased from 0.974 in the control sample to 0.953 in SSIC50, with statistically significant differences (*p* < 0.05) between samples. The reduction in water activity could be due to the high dietary fiber content of the flour.

Thus, coffee silverskin contains a significant proportion of insoluble dietary fiber, which possesses a high water-holding capacity [41], increasing the bound water. Furthermore, replacing fat with hydrophilic flour particles increases the concentration of total solids capable of interacting with the aqueous phase. Finally, the presence of proteins from coffee silverskin flour produces a dense molecular network that restricts molecular mobility and lowers the vapor pressure of the mix, resulting in the observed decrease in water activity. Regarding the pH values of the control and reformulated samples (Table 4), when the amount of coffee silverskin flour added to replace the fat was increased, the pH values decreased (*p* < 0.05), and did so in a concentration-dependent manner. This behavior is consistent with that reported by Khalil et al. [32] and Dang-Bao et al. [42], who showed that low-fat ice cream added with sweet lupine flour or durian-rind-derived low methoxyl pectin, as fat replacers, produced a reduction in pH values. Nevertheless, the pH values were within the characteristic range described for ice-creams (6.3–6.7).

Color is the first physicochemical attribute perceived by consumers and represents one of the main quality indicators of ice cream. In this sense, color parameters strongly affect product marketability and consumer acceptance [43]. In this context, the incorporation of silverskin flour as a partial fat replacer in ice cream formulations produced significant modifications (*p* < 0.05) in all color parameters analyzed (Table 4). In reference to lightness (L*), the control sample showed the highest (*p* < 0.05) values, which is characteristic of conventional ice cream systems rich in fat, where light scattering is primarily determined by fat globules and casein micelles dispersed within the aqueous matrix [1]. Nevertheless, the progressive replacement of cream with silverskin flour significantly (*p* < 0.05) reduced L* values to 54.94 and 46.64 in SSIC25 and SSIC50, respectively, indicating a marked darkening of the product matrix. This reduction in lightness can be attributed to the intrinsic brown coloration from silverskin flour, which originates from the roasting process carried out during coffee production and is associated with the presence of Maillard reaction products, melanoidins, and other thermally generated pigments [44]. Furthermore, the decrease in fat content may have contributed to lower light reflectance, since milk fat globules play a critical role in enhancing opacity and brightness in frozen dairy matrices [45]. Regarding red-green coordinates (a*), a significant increase (*p* < 0.05) was obtained with the replacement of fat with coffee silverskin. While the control ice cream had negative a* values (−1.58), SSIC25 and SSIC50 showed positive values of 4.02 and 4.97, respectively, without statistical differences (*p* > 0.05) between them, suggesting a transition toward red-brown chromatic characteristics. In the same way, a similar trend was observed regarding the yellow-blue coordinate (b*), which increased significantly (*p* < 0.05) from 9.33 in the control ice cream to approximately 14.2 and 14.6 in SSIC25 and SSIC50, respectively, without statistical differences (*p* > 0.05) between them. The increase in both redness and yellowness coordinates indicates that silverskin flour promoted the development of a brownish coloration that is typical of coffee-derived ingredients. With regard to the chroma index (C*), the application of coffee silverskin as a fat substitution in ice cream increases (*p* < 0.05) this parameter from 9.47 in the control sample to 14.76 and 15.45 in SSIC25 and SSIC50, respectively. Chroma is associated with color intensity and saturation; thus, the observed increase demonstrates that the reformulated ice creams exhibited more intense and marked coloration compared with the conventional formulation. On the other hand, the hue (h) values decreased significantly (*p* < 0.05) from 99.74 in the control to 74.09 and 71.16 in SSIC25 and SSIC50, respectively, indicating a substantial shift from yellowish-white tones toward darker orange-brown chromatic regions. This reduction confirms the strong impact of silverskin flour on the optical characteristics of the frozen matrix. Regarding color differences (ΔE), the values obtained for SSIC25 (28.79) and SSIC50 (37.10) were significantly higher than 3 units, which is generally considered perceptible by consumers [46]. The significantly (*p* < 0.05) higher ΔE observed in SSIC50 relative to SSIC25 suggests a concentration-dependent effect, where increasing levels of flour incorporation intensified the deviation from the control formulation.

With reference to the browning index (Table 4), the reformulation of ice creams with coffee silverskin significantly increased (*p* < 0.05) this index from 0.27 in the control to 5.84 and 8.85 in SSIC25 and SSIC50, respectively. Therefore, the marked increase in the browning index confirms that silverskin flour substantially intensified the brown coloration of the ice cream formulations. This effect is closely related not only to the intrinsic color of the ingredient itself but also to the presence of high-molecular-weight melanoidins generated during coffee roasting, compounds that are known to possess strong chromophoric properties and antioxidant activity [47].

The changes observed in all color parameters are commonly reported when flours or powder obtained from fruits are used as fat replacers in ice cream formulations, or when the aim is to enhance the profile of bioactive compounds and improve the overall health-promoting properties of the product. Thus, Yosefiyan et al. [43] informed that the addition of freeze-dried persimmon peel to develop a functional ice cream modified all color parameters. Similarly, Chakraborty et al. [6] mentioned that the use of pomelo (*Citrus maxima*) seed powder as a functional ingredient in low-fat ice cream produced significant color differences with respect to the control in terms of lightness, whiteness, and yellowness parameters.

The overrun of the control ice cream and formulations in which cream was partially replaced by coffee silverskin flour are presented in Table 4. Overrun showed a clear increasing trend (*p* < 0.05) with the incorporation of coffee silverskin flour, rising from 29.207% in the control sample to 38.22% in SSIC25 and reaching 52.17% in SSIC50. These results indicated that a higher level of substitution markedly enhanced the ability of the mixes to incorporate and stabilize air. This behavior may be attributed to the functional properties of coffee silverskin flour, particularly its dietary fiber fraction, which might increase mix viscosity and improve the stabilization of air bubbles during freezing due to the formation of hydrated polysaccharide networks, as mentioned by Soukoulis et al. [48]. In this sense, Masselot et al. [49] reported that ice cream matrices with high viscosity are associated with smaller and more uniformly distributed air bubbles, resulting from reduced coalescence and improved resistance to structural collapse during ice crystal growth. On the other hand, the partial removal of fat may also have reduced destabilizing effects associated with fat globule aggregation, facilitating air incorporation at higher replacement levels [50]. The results obtained agree with those reported by Ghaedrahmati et al. [51] who showed that replacing milk cream with watermelon exocarp powder at 20% and 40% increased the overrun (%) compared with the control ice cream. Similarly, Utpott et al. [52] reported that the use of red pitaya fiber as a fat substitute in ice cream increased the overrun value by 44% compared with the control sample.

The melting behavior of ice cream is influenced by several structural and compositional factors, including total solids content, ice crystal size, emulsifier functionality, protein concentration, and thermal diffusivity [53]. These parameters directly affect the stability of the ice cream matrix and its resistance to melting during storage and consumption [54]. Table 4 shows the melting rate values of the control ice cream and reformulated ice creams added with coffee silverskin flour. As can be seen, the substitution of cream with coffee silverskin flour produced a slight increase in this parameter. However, no statistically significant differences were found (*p* > 0.05) between formulations. These results suggest that fat substitution up to 31.56% did not significantly compromise resistance to melting. Previous studies mentioned in the literature show that low contents of fat increase the melting rate as a result of an increase in thermal diffusivity, since cream has a protective effect on ice cream against heat diffusion [55,56]. In addition, higher overrun could reduce the meltdown rate due to influencing the thermal diffusivity, as mentioned by Akbari et al. [57]. However, as occurred in the study carried out by Utpott et al. [52] and in this work, the increase in overrun did not have a statistically significant effect on melting rate.

### 3.3. Aroma Profile

The addition of coffee silverskin flour as a partial fat replacer significantly modified the volatile aromatic profile of the ice cream formulations (Table 5). Therefore, twenty-seven volatile compounds belonging to several chemical families, including aldehydes, ketones, pyrazines, terpenes, esters, lactones, alcohols, and furans, were identified in the control, SSIC25, and SSIC50 samples.

The control sample was mainly characterized by high concentrations of ketones, particularly 2-heptanone and 2-nonanone, which represented the predominant volatile compounds in the formulation. These compounds are commonly associated with dairy products and are responsible for fatty, fruity, and blue-cheese-like sensory notes [59]. The high abundance of these ketones is consistent with the elevated milk fat content of the control ice cream, since methyl ketones are typically generated through β-oxidation and thermal degradation of fatty acids during processing and storage [60]. In addition, the control sample exhibited higher levels of hexanal and D-limonene, contributing green, grassy, and citrus-like notes to the aroma profile. Hexanal is frequently considered a marker of lipid oxidation in dairy matrices and is strongly associated with the oxidation of linoleic-acid-derived compounds [61]. The partial substitution of fat with coffee silverskin flour resulted in a progressive decrease in ketone-derived compounds. The relative abundance of 2-heptanone decreased from 33.70% in the control sample to 20.07% and 13.07% in SSIC25 and SSIC50, respectively. Similarly, 2-nonanone decreased from 21.32% in the control to 13.01% and 8.54% in the reformulated samples. This reduction can be attributed to the lower fat content of the formulations, since lipid-derived ketones are directly related to the availability of fatty acid precursors. The reduction in these compounds may contribute to a lower intensity of traditional dairy and creamy aroma notes in the reformulated ice creams.

On the other hand, the use of coffee silverskin flour as a fat replacer facilitated the formation of compounds associated with roasted, nutty, and caramel-like sensory attributes. Therefore, tetramethylpyrazine, which was absent in the control sample, reached values of 9.01% and 11.10% in SSIC25 and SSIC50, respectively. Pyrazines are well-known Maillard reaction products commonly found in roasted coffee and thermally processed foods, and they are strongly associated with cocoa, toasted, and nutty aromas [62]. Likewise, 4,6-dimethylpyrimidine is found to appear solely in ice creams where the fat is substituted, further supporting the contribution of coffee silverskins to the aromatic complexity of reformulated samples. The presence of furfural in SSIC25 and SSIC50 also indicates the contribution of thermally generated compounds originating from the coffee silverskin flour [63]. This compound is typically produced through carbohydrate degradation and Maillard reactions and is associated with sweet, caramel, and baked sensory descriptors [64]. Although detected at relatively low concentrations, its presence may significantly contribute to the overall aroma perception due to its low odor threshold.

Aldehydes also showed notable differences among formulations. Benzaldehyde was absent in the control sample but increased considerably in SSIC25 and SSIC50, reaching values of 5.76% and 6.49%, respectively. This compound is associated with almond and cherry-like notes and may contribute positively to the sensory complexity of the reformulated products. Similarly, 3-methylbutanal was detected exclusively in SSIC50, providing malty, chocolate-like, and nutty notes that are highly compatible with coffee-derived aromatic characteristics. The formation of Strecker aldehydes such as 3-methylbutanal is commonly linked to Maillard reaction pathways involving amino acid degradation during thermal processing [65].

The reformulated ice creams also exhibited an increase in terpene- and terpenoid-derived compounds. Linalool oxide, not detected in the control sample, increased to 5.02% and 7.08% in SSIC25 and SSIC50, respectively, while β-caryophyllene was identified only in SSIC25. Despite the reduction in fat-derived volatiles, lactones such as δ-decalactone and δ-nonalactone remained relatively stable among formulations. Lactones are associated with creamy and dairy aromas and are considered important contributors to the characteristic flavor of ice cream [66]. Their stability suggests that the partial replacement of fat with coffee silverskin flour did not completely compromise the creamy aroma associated with dairy desserts.

The increase in aromatic diversity observed in SSIC25 and SSIC50 indicates that coffee silverskin flour acts not only as a fat replacer but also as a source of aroma-active compounds capable of modifying the sensory profile of ice cream. In particular, the SSIC50 formulation showed the highest abundance of roasted and Maillard-derived volatiles, suggesting a stronger coffee-associated aromatic profile.

### 3.4. Polyphenolic Profile and Caffeine Content

The incorporation of silverskin flour notably influenced the functional composition of the reformulated ice creams, particularly regarding the enrichment of bioactive compounds naturally present in this coffee bean roasting coproduct. Thus, coffee silverskin is a vast source of bioactive compounds, including polyphenolic compounds and methylxanthines [14,67].

**Figure 2:**
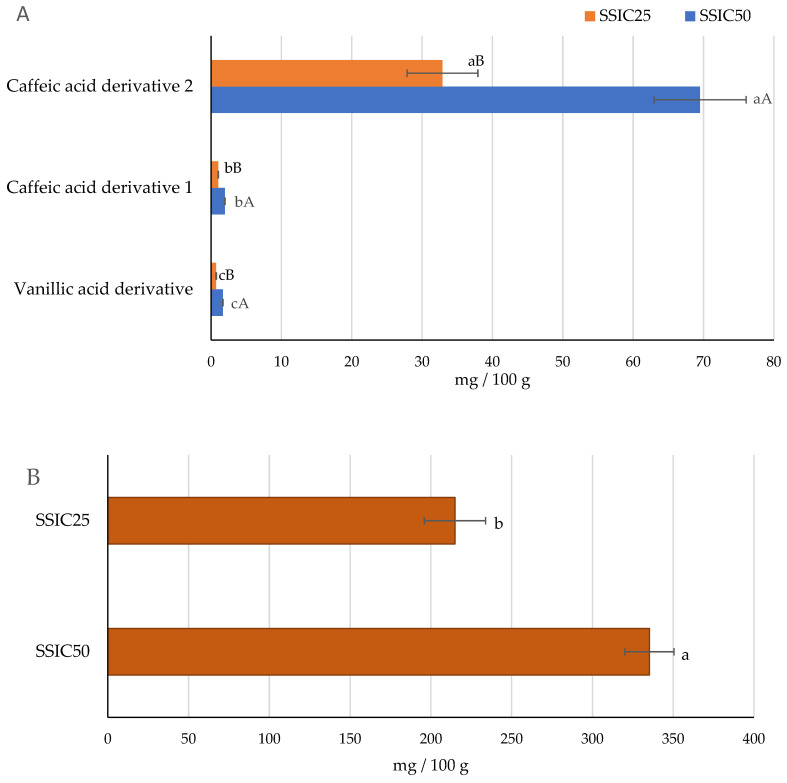
(**A**) Polyphenolic profile; (**B**) caffeine content of reformulated ice creams added with coffee silverskin flour. SSIC25: ice cream with 15.78% cream replaced by coffee silverskin flour; SSIC50: ice cream with 31.56% cream replaced by coffee silverskin flour. In the polyphenolic profile (**A**), for each compound, histograms with the same capital letter indicate that there are no statistically significant differences (*p* > 0.05) according to Tukey’s multiple range test. For each sample (SSIC25 and SSIC50), histograms with the same small letter indicate that there are no statistically significant differences (*p* > 0.05) according to Tukey’s multiple range test. Regarding caffeine content (**B**), histograms with the same small letter indicate that there are no statistically significant differences (*p* > 0.05) according to Tukey’s multiple range test.

The polyphenolic profile of ice creams in which fat was partially replaced by coffee silverskin flour is present in Figure 2A. As expected, no polyphenolic compounds were detected in the control sample. In contrast, only three compounds were identified in the reformulated ice creams (SSIC25 and SSIC50): two caffeic acid derivatives and one vanillic acid derivative. For all identified compounds, concentrations were significantly higher (*p* < 0.05) in SSIC50 than in SSIC25.

Among the analyzed compounds, caffeic acid derivative 2 was the predominant phenolic constituent in both ice cream samples (*p* < 0.05). SSIC50 exhibited a markedly higher concentration, reaching 69.51 mg/100 g, whereas SSIC25 showed values of 32.90 mg/100 g. On the other hand, caffeic acid derivative 1 and vanillic acid derivative were detected at considerably lower concentrations in both formulations, remaining below 2 mg/100 g. Nevertheless, a slight increase was observed in SSIC50 relative to SSIC25 (*p* < 0.05), reinforcing the hypothesis that phenolic enrichment is concentration-dependent.

Figure 2B shows the caffeine content of ice cream formulations in which fat was partially replaced by coffee silverskin flour. SSIC25 had a caffeine content of 214.90 mg/100 g, whereas SSIC50 showed a considerably higher value (335.25 mg/100 g), with statistically significant differences (*p* < 0.05) between samples. No caffeine was detected in the control sample.

The markedly higher caffeine content observed in SSIC25 and SSIC50 ice cream is attributed to the addition of silverskin flour, which directly increases the caffeine concentration in the ice cream matrix. Since caffeine is relatively stable under common food-processing conditions, most of the compound is expected to remain intact during formulation and freezing [68].

These values are consistent with those reported by Bertolino et al. [69] who found that the caffeine concentration in cow whole-milk yogurt added with coffee silverskin at 2%, 4%, or 6% ranged between 173 and 335 mg/100 g. Similarly, Guglielmetti et al. [70] reported that gluten-free bread added with coffee silverskin flour at 2.5% had a caffeine content of 72 mg/100 g. Caffeine is one of the main bioactive compounds present in coffee silverskin. Caffeine is widely recognized for its numerous positive health effects, such as neurostimulation, promotion of vasodilation and diuresis, and reduction in the progression of Parkinson’s disease and cognitive disorders, as well as possessing anti-inflammatory and anticarcinogenic properties, while also supporting cardiovascular health [71]. However, it is important to highlight that, according to the European Food Safety Authority, caffeine intake should not exceed 400 mg/day for the general population and 200 mg/day for pregnant women [72]. In this context, formulations containing high levels of coffee-derived ingredients may require appropriate labeling and dosage evaluation to ensure consumer safety.

### 3.5. Sensory Analysis

The results of the hedonic sensory analysis are shown in Figure 2A. The scores obtained for the ice cream samples showed an inverse relationship between the concentration of coffee silverskin flour added to replace the fat content and consumer liking across all sensory dimensions. The control sample maintained the highest scores (*p* < 0.05) across all categories except aroma, between 7.0 and 8.0. As the substitution level increased, the sensory attributes of the samples declined, with SSIC50 exhibiting the most significant reduction across all parameters, except for aroma.

This parameter, aroma, was the attribute least affected by the inclusion of coffee silverskin. The control sample scored 6.17, while both SSIC25 and SSIC50 remained closely grouped with values of 6.24 and 6.74, respectively. This suggests that the volatile compounds characteristic of coffee silverskin, which are associated with the Maillard reaction during roasting, provide a pleasant, toasted olfactory profile that consumers find acceptable even at higher replacement levels. On the other hand, color scores showed a marked decline with the substitution of fat with coffee silverskin. The control sample score (7.61) dropped to 6.24 and 5.98 for SSIC50 and SSIC25, respectively. The incorporation of coffee silverskin flour imparts a dark brown hue to the ice cream matrix, which deviates significantly from the control sample, contributing to lower consumer acceptability ratings.

The most substantial hedonic changes occurred in the flavor-related attributes. Taste and sweetness scores for the control sample were robust, with values of 7.26 and 7.53, respectively. However, the replacement of fat with coffee silverskin produced a deep reduction in the scores obtained. Thus, SSIC50 had values for these two parameters close to 5.0 (“neither like nor dislike”). The decline in sweetness is particularly noteworthy. While the actual sugar content remained constant across samples, the presence of coffee silverskin appears to have a masking effect. This could be attributed to the increase in bitterness, where liking scores dropped from 7.2 (the control sample) to 5.15 (SSIC50). Coffee silverskin had caffeine and (poly)phenolic compounds in its composition, which could introduce these bitter notes. Furthermore, the reduction in fat (a flavor carrier) in the SSIC50 sample likely resulted in a faster release of bitter compounds and less flavor balance. For grittiness, again the control sample showed the highest value with a score of 7.71, while SSIC50 exhibited the lowest liking score in this category, with a value of 5.14. Coffee silverskin is highly fibrous. Even when processed into a fine flour, the insoluble fiber particles may create a “sandiness” in the mouth. In an ice cream matrix, where smoothness is a primary quality indicator, these particles interfere with the perception of creaminess. Furthermore, the reduction in fat in the SSIC50 sample means that there is less fat to lubricate these fiber particles, exacerbating the perception of a coarse texture.

**Figure 3:**
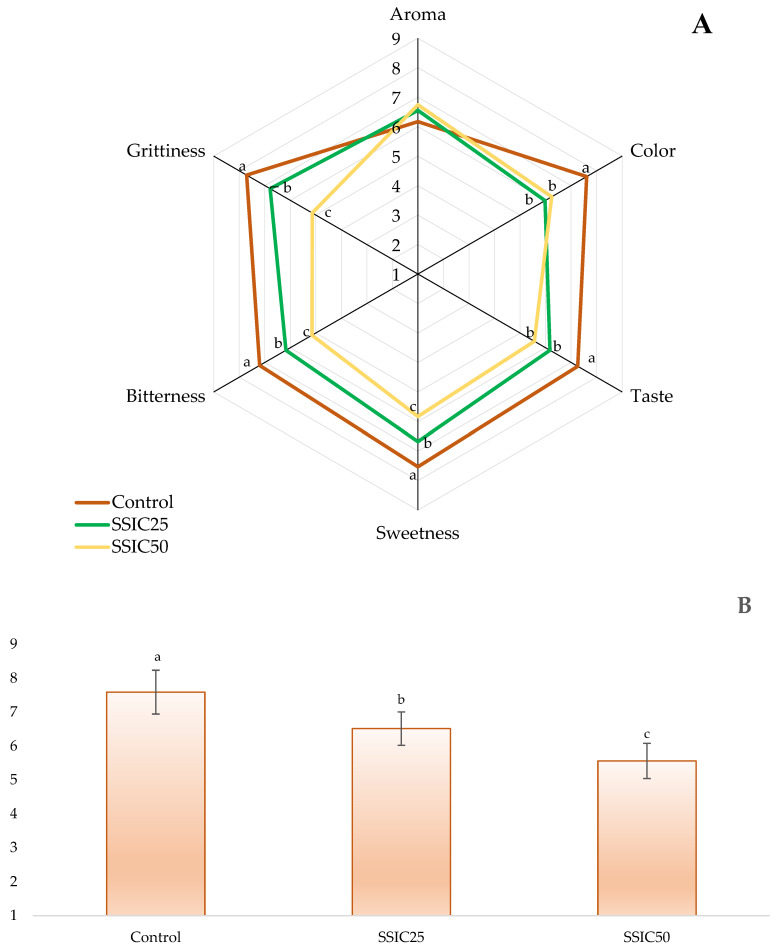
Sensory evaluation (**A**) and overall acceptance (**B**) of the control ice cream and reformulated ice creams added with coffee silverskin flour. SSIC25: ice cream with 15.78% cream replaced by silverskin flour; SSIC50: ice cream with 31.56% cream replaced by silverskin flour. In sensory evaluation, for the same attribute, lines with the same small letter indicate that there are no statistically significant differences (*p* > 0.05) according to Tukey’s multiple range test. In overall acceptance, histograms with the same small letter indicate that there are no statistically significant differences (*p* > 0.05) according to Tukey’s multiple range test.

In terms of overall acceptance (Figure 3B), scores indicated a dose-dependent decline following the addition of coffee silverskin flour. The control sample achieved the highest mean score (7.59), whereas the SSIC25 and SSIC50 formulations showed progressively lower values of approximately 6.51 and 5.56, respectively, with statistically significant differences (*p* < 0.05) between samples. This trend suggests that higher substitution levels of coffee silverskin significantly impact the overall sensory profile and consumer preference for the ice cream. Although the present study identified 15.78% as the highest substitution level which maintained acceptable consumer scores, several formulation strategies could be explored to mitigate these sensory drawbacks. Reducing the particle size of the coffee silverskin flour or applying micronization treatments could minimize grittiness. Bitterness could be balanced by optimizing the sweetener system, incorporating natural flavor modulators such as vanilla or cocoa, or blending coffee silverskin with other neutral fiber sources. Furthermore, future studies employing response surface methodology or mixture design could optimize the balance between nutritional enhancement and sensory acceptance, allowing the identification of formulations with improved consumer acceptability while preserving the functional benefits of coffee silverskin.

## 4. Conclusions

The results of the present study demonstrated that replacing fat with an industrial coproduct such as coffee silverskin can improve the nutritional profile of ice creams while contributing to the valorization of waste generated by the coffee industry, which promotes circular economy principles.

The findings indicate that silverskin flour is an effective fat replacer and simultaneously enhances the concentration of phenolic compounds in ice cream formulations. Furthermore, the higher caffeine content may provide an additional functional characteristic for consumers interested in products with stimulant or energizing effects. Additionally, the use of coffee silverskin increased the dietary fiber and mineral content. This combined functionality underscores the potential of coffee-derived coproducts as sustainable ingredients for the development of functional foods with improved nutritional value.

From a sensory perspective, the replacement of fat by coffee silverskin flour at different concentrations produced significant changes in the relative abundance of key aroma-active compounds, resulting in the development of a more complex and roasted aromatic profile compared with the control formulation. In addition, the results suggest that a 15.78% replacement level (SSIC25) represents the maximum threshold for maintaining favorable consumer acceptance (scores > 6.0). Above this level, the combined increase in bitterness and perceived grittiness negatively affects overall sensory acceptance. Future research should investigate the bioavailability of bioactive compounds, the long-term stability of the formulations, and consumer acceptance in larger and more diverse populations.

## Figures and Tables

**Table 1:** Formulations of control ice cream and ice creams added with coffee silverskin flour as fat replacers.

	^1^ Control	SSIC25	SSIC50
Whole milk	58	58	58
Milk cream	19	16	13
Skimmed milk powder	4.5	4.5	4.5
Sugar	15	15	15
Inulin	3	3	3
Soy lecithin	0.5	0.5	0.5
Coffee silverskin flour	0	3	6
Replacement level (%)	---	15.78	31.56

^1^ Values expressed as g/100 g. SSIC25: ice cream with 15.78% cream replaced by coffee silverskin flour; SSIC50: ice cream with 31.56% cream replaced by coffee silverskin flour.

**Table 2:** Chemical composition of the control ice cream and reformulated ice creams added with coffee silverskin flour as fat replacers.

	Moisture ^1^	Fat	Protein	Ash	Dietary Fiber
Control	61.89 ± 0.42 ^a^	10.14 ± 0.12 ^a^	3.55 ± 0.10 ^c^	0.87 ± 0.09 ^c^	3.22 ± 0.12 ^c^
SSIC25	60.13 ± 0.52 ^b^	8.00 ± 0.36 ^b^	4.18 ± 0.07 ^b^	1.20 ± 0.01 ^b^	5.18 ± 0.23 ^b^
SSIC50	58.15 ± 0.17 ^c^	5.91 ± 0.27 ^c^	4.72 ± 0.03 ^a^	1.36 ± 0.05 ^a^	7.34 ± 0.18 ^a^

^1^ Values are expressed in g/100 g of ice cream as mean ± standard deviation of three independent replicates (n = 3). SSIC25: ice cream with 15.78% cream replaced by coffee silverskin flour; SSIC50: ice cream with 31.56% cream replaced by coffee silverskin flour. Values followed by the same letter within the same column indicate that there are no statistically significant differences (*p* > 0.05) according to Tukey’s multiple range test.

**Table 3:** Mineral profiles of the control and reformulated ice creams added with coffee silverskin flour.

	Control	SSIC25	% Increase	SSIC50	% Increase
Calcium ^1^	167.76 ± 8.97 ^b^	207.78 ± 12.48 ^a^	23.85	214.35 ± 9.16 ^a^	27.77
Cupper	0.01 ± 0.00 ^c^	0.24 ± 0.01 ^b^	2300	0.43 ± 0.01 ^a^	4200
Iron	0.16 ± 0.01 ^c^	1.03 ± 0.05 ^b^	543.27	1.84 ± 0.02 ^a^	1050
Potassium	203.46 ± 5.82 ^c^	271.95 ± 2.08 ^b^	33.66	281.92 ± 1.58 ^a^	38.56
Magnesium	16.93 ± 1.15 ^c^	27.61 ± 1.62 ^b^	63.08	30.04 ± 0.35 ^a^	77.43
Sodium	54.07 ± 1.55 ^a^	52.89 ± 1.75 ^a^	−2.18	42.47 ± 2.18 ^b^	−21.45
Phosphorus	142.18 ± 1.55 ^a^	139.78 ± 2.34 ^a^	−1.69	114.51 ± 2.19 ^b^	−19.46
Zinc	0.61 ± 0.03 ^b^	0.69 ± 0.01 ^a^	13.11	0.73 ± 0.02 ^a^	19.67%

^1^ Values are expressed in mg/100 g of ice cream as mean ± standard deviation of three independent replicates (n = 3). SSIC25: ice cream with 15.78% cream replaced by coffee silverskin flour; SSIC50: ice cream with 31.56% cream replaced by coffee silverskin flour. Values followed by the same letter within the same row indicate that there are no statistically significant differences (*p* > 0.05) according to Tukey’s multiple range test.

**Table 4:** The physicochemical properties of the control ice cream and reformulated ice creams added with coffee silverskin flour as fat replacers.

	^1^ Control	SSIC25	SSIC50
Aw	0.974 ± 0.011 ^a^	0.962 ± 0.004 ^b^	0.953 ± 0.002 ^c^
pH	6.56 ± 0.03 ^a^	6.37 ± 0.03 ^b^	6.20 ± 0.01 ^c^
L*	82.71 ± 1.01 ^a^	54.94 ± 1.81 ^b^	46.64 ± 0.71 ^c^
a*	−1.58 ± 0.04 ^b^	4.02 ± 0.45 ^a^	4.97 ± 0.45 ^a^
b*	9.33 ± 1.18 ^b^	14.19 ± 1.50 ^a^	14.62 ± 1.65 ^a^
C*	9.47 ± 1.16 ^b^	14.76 ± 2.19 ^a^	15.45 ± 1.70 ^a^
h	99.74 ± 1.20 ^a^	74.09 ± 3.48 ^b^	71.16 ± 0.88 ^c^
ΔE	---	28.79 ± 2.30 ^b^	37.10 ± 1.79 ^a^
BI	0.27 ± 0.16 ^c^	5.84 ± 0.67 ^b^	8.85 ± 0.59 ^a^
Overrun (%)	29.2 ± 0.97 ^c^	38.22 ± 0.79 ^b^	52.17 ± 0.69 ^a^
Melting rate (g/min)	0.57 ± 0.03 ^a^	0.59 ± 0.03 ^a^	0.62 ± 0.01 ^a^

Values are expressed as mean ± standard deviation of three independent replicates (n = 3). ^1^ SSIC25: ice cream with 15.78% cream replaced by coffee silverskin flour; SSIC50: ice cream with 31.56% cream replaced by coffee silverskin flour. Values followed by the same letter within the same row indicate that there are no statistically significant differences (*p* > 0.05) according to Tukey’s multiple range test.

**Table 5:** Aroma profiles of the control ice cream and reformulated ice creams added with coffee silverskin flour.

Compound	Ret. Index (lit.)	Ret. Index (exp.)	Control Area (%)	SSIC25 Area (%)	SSIC50 Area (%)	Chemical Family	Sensory Descriptors
3-Methylbutanal	655	658	0.00	0.00	7.01 ^a^	Aldehyde	Malty, chocolate, nutty
Hexanal	800	802	12.01 ^a^	10.36 ^b^	8.61 ^c^	Aldehyde	Green, grassy, leafy
Furfural	826	827	0.00	0.67 ^b^	0.80 ^a^	Furan	Sweet, caramel, baked
Ethyl 2-methylbutanoate	835	834	2.46 ^a^	0.68 ^b^	0.35 ^c^	Ester	Fruity, apple, pineapple
2-Heptanone	890	894	33.98 ^a^	20.07 ^b^	13.15 ^c^	Ketone	Blue cheese, fatty, fruity
4,6-Dimethylpyrimidine	906	908	0.00	2.45 ^b^	3.85 ^a^	Pyrimidine	Roasted, toasted, nutty
Benzaldehyde	961	960	0.00	5.76 ^b^	6.53 ^a^	Aldehyde	Almond, cherry
D-Limonene	1028	1034	6.02 ^a^	3.08 ^c^	4.20 ^b^	Terpene	Citrus, orange peel
Benzyl alcohol	1034	1036	0.00	0.60 ^b^	1.15 ^a^	Alcohol	Floral, sweet
(E)-2-Octenal	1062	1058	1.44 ^b^	2.87 ^a^	2.85 ^a^	Aldehyde	Fatty, cardboard, green
Linalool oxide	1074	1072	0.00	5.02 ^b^	7.13 ^a^	Terpenoid	Woody, herbal, floral
Tetramethylpyrazine	1088	1080	0.00	9.01 ^b^	11.17 ^a^	Pyrazine	Roasted, cocoa, nutty
2-Nonanone	1090	1088	21.49 ^a^	13.01 ^b^	8.59 ^c^	Ketone	Fatty, dairy, fruity
Nonanal	1102	1104	3.15 ^c^	7.06 ^a^	5.14 ^b^	Aldehyde	Fatty, citrus peel
2-Nonenal	1160	1162	0.00	0.00	2.10 ^a^	Alkene	Green, cucumber
Ketoisophorone	1166	1170	0.00	0.00	0.19 ^a^	Ketone	Caramel, cooked
p-Methylacetophenone	1183	1184	0.00	0.69 ^b^	1.21 ^a^	Ketone	Sweet, floral, honey
Dodecane	1200	1205	9.23 ^a^	8.86 ^b^	8.25 ^c^	Alkane	Waxy, oily
Decanal	1205	1208	0.76 ^c^	1.26 ^a^	0.99 ^b^	Aldehyde	Citrus, fatty, soapy
γ-Octalactone	1260	1258	0.92 ^b^	0.98 ^a^	1.01 ^a^	Lactone	Coconut, dairy
2-Undecanone	1291	1294	5.14 ^a^	3.82 ^b^	3.11 ^c^	Ketone	Herbal, green
Triacetin	1305	1304	0.47 ^b^	1.05 ^a^	0.35 ^c^	Ester	Mild sweet, neutral
β-Caryophyllene	1418	1414	0.00	0.53 ^a^	0.00	Terpene	Woody, spicy
(E)-α-Bergamotene	1436	1435	0.23 ^a^	0.00	0.00	Terpene	Citrus, woody
δ-Decalactone	1490	1488	1.05 ^c^	1.33 ^a^	1.23 ^b^	Lactone	Peach, creamy, dairy
2-Tridecanone	1496	1498	0.82 ^a^	0.85 ^a^	0.73 ^b^	Ketone	Green, fatty
β-Bisabolene	1509	1510	0.31 ^a^	0.00	0.00	Terpene	Sweet, balsamic, floral

Mean value of three independent replicates (n = 3). Retention indices: exp. = experimental values; lit. = literature values (NIST Spectra Database). Sensory descriptors were obtained from The Good Scents Company database [58]. Values followed by the same letter within the same row indicate that there are no statistically significant differences (*p* > 0.05) according to Tukey’s multiple range test.

## Data Availability

The original contributions presented in this study are included in the article. Further inquiries can be directed to the corresponding author.
